# Complete Genome Sequence of Vancomycin-Intermediate *Staphylococcus aureus* Strain MI (HIP5827)

**DOI:** 10.1128/genomeA.00123-16

**Published:** 2016-03-17

**Authors:** Tomomi Hishinuma, Yuki Katayama, Miki Matsuo, Takashi Sasaki, Keiichi Hiramatsu

**Affiliations:** a Department of Microbiology, Faculty of Medicine, Juntendo University, Bunkyo-ku, Tokyo, Japan; b Research Centre for Infection Control Science, Juntendo University, Bunkyo-ku, Tokyo, Japan

## Abstract

We report the complete genome sequence of vancomycin-intermediate *Staphylococcus aureus* (VISA) strain MI (HIP5827).

## GENOME ANNOUNCEMENT

HIP5827 is a vancomycin-intermediate *Staphylococcus aureus* (VISA) clinical isolate from the United States (MI), exhibiting reduced susceptibility to vancomycin (MIC = 8 mg/L) (1). The biological properties of HIP5827 are different from those of strain Mu50, the first documented VISA strain in the world (2). Compared to Mu50, HIP5827 did not have a thick cell wall (3, 4), however, the autolytic activity was significantly decreased (unpublished data). Determination of the whole-genome sequence of HIP5827 was done to explore a novel mechanism of vancomycin resistance.

The genome of strain MI was sequenced using a PacBio RS II sequencer (10 kbp insert library; average read length 8,259 bp; 127,906 reads; average depth 369) and an Illumina Miseq (300-mer paired-end; 543,770 × 2 reads). *de novo* assembly using HGAP3 (PacBio DevNet, Pacific Biosciences, Menlo Park, CA) produced two circular contigs composed of a chromosome and a plasmid. To correct sequencing errors, we mapped the Illumina reads to the PacBio contigs and built consensus sequences using bwa-0.7.5a and samtools-0.1.19 (5, 6). Gene annotation was performed on the Rapid Annotations using Subsystems Technology (RAST) server (7) and the KEGG Automatic Annotation Server (KAAS) (8).

The MI chromosomal and plasmid genomes are 2,860,370 bp (G+C content, 32.9 %) and 55,980 bp (G+C content, 29.5 %) in size, respectively. The chromosomal genome possesses 2,684 open reading frames, 5 ribosomal operons, and 56 tRNAs. There are 62 protein-coding sequences on the plasmid.

This entry of GenBank on the strain of *S. aureus* MI will contribute to various research fields in addition to antimicrobial resistance, such as physiology, genome evolution, and epidemiology.

### Nucleotide sequence accession numbers.

The complete genome sequence of *S. aureus* strain MI has been deposited in the GenBank database under the accession numbers AP017320 and AP017321 for the chromosome and plasmid, respectively.
